# Relationship between the Apolipoprotein AI, B gene polymorphism and the risk of non-traumatic osteonecrosis

**DOI:** 10.1186/1476-511X-13-149

**Published:** 2014-09-23

**Authors:** Ji-Min Yin, Zhao Liu, Shi-Chang Zhao, Yan-Jie Guo, Zhong-Tang Liu

**Affiliations:** Department of Orthopedic Surgery, Shanghai Sixth People’s Hospital, Shanghai Jiaotong University, No.600 Yishan Road, Shanghai, 200233 China; Department of hematology, Shanghai Rui Jin Hospital, Shanghai Jiao Tong University School of Medicine, Shanghai, China

**Keywords:** Apolipoprotein AI, Apolipoprotein B, Non-traumatic osteonecrosis, Gene polymorphism, Molecular epidemiology

## Abstract

**Background:**

Previous studies suggested that Apolipoprotein AI (ApoAI) and apolipoprotein B (ApoB) gene polymorphisms may result in lipid metabolism disorders. Genetic polymorphisms in these genes may be associated with the occurrence of osteonecrosis.

**Methods:**

We designed a case-control study including 429 patients of osteonecrosis and 368 age- and sex-matched control subjects. Polymerase chain reaction was used to amplify the DNA fragments in promoter -75 G > A of ApoAI gene and EcoR I, Xba I and 3′-VNTR of ApoB gene in osteonecrosis patients and healthy controls. We utilized polymerase chain reaction-restriction fragment length polymorphism (PCR-RFLP) method to genotype these four single nucleotide polymorphisms (SNPs).

**Results:**

For -75 G > A polymorphism of ApoAI, AA genotype frequency (0.501) was significantly higher in patients with osteonecrosis than that in control (0.462) subjects (*P* <0.001), GA genotype frequency (0.170) was significantly lower than that in the control (0.310) group (*P* <0.0001). In osteonecrosis patients, the odds ratio (OR) of A allele was 3.932 (95% CI: 3.0847 ~ 5.0123), which suggested that subjects carrying A allele of promoter region -75 G > A of ApoAI gene had higher susceptibility to osteonecrosis than G allele carriers. The genotype and allele frequency distributions showed no significant difference in EcoR I, Xba Iand 3′-VNTR loci of ApoB gene between the osteonecrosis group and control group.

**Conclusion:**

Our study suggested that ApoAI gene -75G > A polymorphism may be associated with susceptibility to osteonecrosis in Chinese population. However, our results need further investigation with large sample size and various populations.

## Introduction

Osteonecrosis is one kind of orthopedic refractory disease and is divided into traumatic and non-traumatic one. Non-traumatic osteonecrosis, with rapid progression, is common seen in young people [1]. Currently, the whole population of osteonecrosis is about 7 million people in China, and new cases have reached 100-200 thousand each year [2]. Previous studies indicated that the abnormal lipid metabolism is known to be the main pathogenesis of osteonecrosis [3]. Heavy drinking and excessive use of corticosteroids may result in abnormal lipid metabolism in general populations [4]. Therefore, among the etiologies of osteonecrosis, both of them were commonly considered to have the same pathogenesis. Studies showed that the incidence of coronary heart disease (CHD) was associated with abnormal ApoAI and ApoB levels resulting from apoAI and apoB gene polymorphisms [5–7]. However, the relationship between these polymorphisms and non-traumatic osteonecrosis has not been systematically studied. In 2007, Hirata et al. [8] performed a case-control study to reveal the relation between ApoAI and ApoB genetic polymorphisms and osteonecrosis in a Japanese population. In their study, four SNPs including C7623T and G12619A for the ApoB gene and G75A and C83T for the ApoAI gene were analyzed using PCR-RFLP and TaqMan real-time PCR method. The authors found C7623T polymorphism of ApoB gene was associated with osteonecrosis. Subsequently, Wang et al. [9] found -75 G > A polymorphism was associated with osteonecrosis in Chinese population. However, the sample sizes of these two studies were very small.

Up to date, a number of SNPs of ApoAI and ApoB were reported, such as -75 G > A, +75 bp and +83 bp loci of Apo AI gene, Eco RI loci, Xba I loci and 3′-VNTR of Apo B gene, which were reported to be associated with several disease [8–13]. To further clarify the association of ApoAI and ApoB genetic polymorphism and osteonecrosis, we designed a larger sample-size case-control study.

### Subjects and methods

#### Ethics

The present study has been performed with the approval of the ethics committee of Shanghai Jiaotong University and was in compliance with the Helsinki Declaration. The informed consents of the study were collected from all the candidate subjects.

#### Subjects

All the patients were selected from November 2001 to September 2013 in Shanghai Sixth People’s Hospital. All the patients were consistent with the diagnostic criteria of osteonecrosis proposed in 1995 by Mont et al. [14]. They were all confirmed by clinical diagnosis, double hip X-ray image, CT scan or MRI examination. A total of 429 cases including 326 male and 103 female with the average age of 44.6 ± 11.3 years were included. Exclusion criteria: 1) Primary disease in serious condition which required hormone replacement therapy. 2) Drugs use which can affect the lipid metabolism and liver enzyme in patients. 3) It did not meet the diagnostic criteria of osteonecrosis or patients with traumatic osteonecrosis and other hip diseases. 4) Patients who were reluctant to be included in this study.

The subjects in the control group were selected from the same hospital. They did not have osteonecrosis and other related diseases. A total of 368 subjects including 278 male and 90 female were selected as the control group, they were aged 44.7 ± 11.7 years old. They have same exposure situation as the case group in Shanghai Sixth People’s Hospital, and have the associated primary disease and accept the system hormone therapy with a clear hormone amount and time of more than one month, and after a 1-year follow-up, no osteonecrosis was found. Subject with poor compliance, incomplete clinical data and vague diagnosis were excluded.

## Methods

### Blood collection and DNA preparation

Blood samples were collected using a standard venipuncture technique and EDTA-containing tubes.DNA was extracted from peripheral vein blood leukocytes using a whole blood genome extraction kit (Beijing Boiteke Corporation, Beijing, China).

### Primers and genotyping

**Figure 1:**
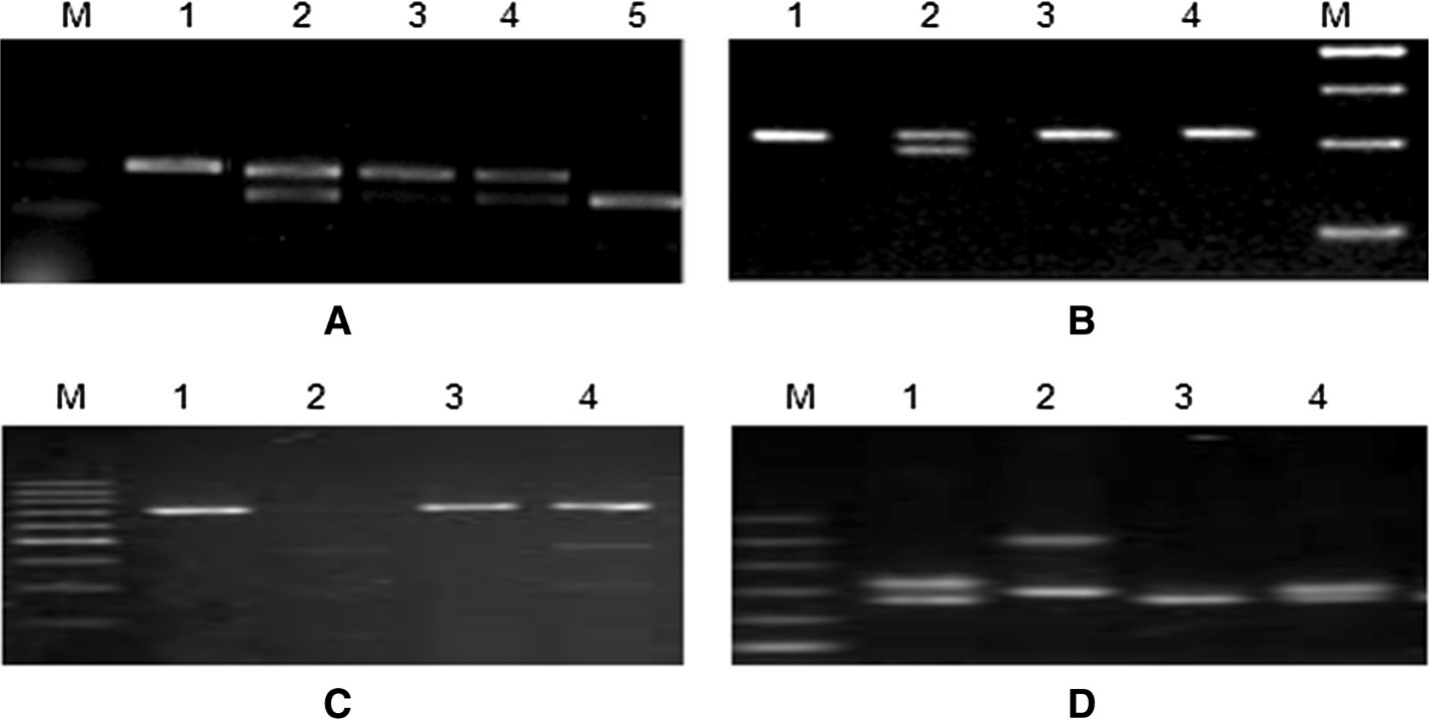
**Gel electrophoresis images by PCR-RFLP method.** The genotyping results: **A**: -75 G > A of ApoAI gene, 1, 3 GG; 2,4: GA, 5: AA. **B**: EcoR I loci of ApoB gene: 1,3,4: E + E+, 2:E + E-; **C**: XbaI loci of ApoB gene: 1, 3: X-X-; 2,4: X + X-; **D**: 3′-VNTR of ApoB gene. 1,2: SB, 3,4: SS.

The primers were designed according to previous literatures [15–19]. All primers were synthesed by Shanghai Sangon Biological Engineering Company (Shanghai China). Genotyping methods were performed according to references [16–19]. Briefly, polymerase chain reaction (PCR) was performed in a volume of 25 ml containing 200 ng genomic DNA. The amounts of Mg^2+^, dNTP, and DNA polymerase (Bangalore Genei, India) used in each reaction were 1.5 mM, 200 mM, and 1 U, respectively. The thermal cycles started with 94°C for 4 min and were followed by 35 cycles of 94°C for 30 s, 55°C for 30 s, and 72°C for 30 s. A total volume of 20 ul containing 20 U endonuclease was added directly to the PCR product and digested at 37°C overnight. After electrophoresis, the digested products were visualized on a 3% polyacrylamide gel with ethidium bromide staining. The genotyping results were shown in Figure 1.

### Statistical analysis

Data were analyzed using SPSS 17.0 software package (Chicago, IL, USA). The genotype and allele frequencies were calculated by direct counting method. The differences of genotype and allele distributions between case and control groups were compared using χ2 test, OR value and its 95% CI was calculated according to logistic regression analysis. Single-SNP effects with continuous variables were analyzed using general linear model (GLM). Normality was assessed by plotting the residuals. Statistical significance was set at p <0.05.

## Results

### Characteristics of participants

The characteristics of 429 patients and 368 control subjects were shown in Table 1. After the statistical analysis, there were no significant differences in the distribution of age, sex, body mass index (BMI), GLU, TC and HDL-C between the two groups. However, there were significant differences in TG and LDL-C between these two groups.

**Table 1:** **Characteristics of the participants**

Groups	N	Age (years)	Gender	BMI Kg/m ^2^	GLU (mmol/L)	TG (mmol/L)	TC (mmol/L)	HDL-C (mmol/L)	LDL-C (mmol/L)
ONFH group	429	44.6 ± 11.3	326/103	24.6 ± 3.8	5.4 ± 1.6	2.1 ± 1.0	4.8 ± 2.5	1.5 ± 0.9	2.7 ± 1.4
Control group	368	44.7 ± 11.7	278/90	24.9 ± 3.7	5.3 ± 1.5	1.7 ± 1.1	4.7 ± 1.9	1.4 ± 0.7	2.4 ± 1.6
*P*		0.903	0.949	0.261	0.352	<0.001	0.531	0.084	0.005

Note: BMI = Body mass index; GLU = Glucose; TC = Total cholesterol; LDL-C = Low-density lipoprotein-cholesterol; LDL-C = High-density lipoprotein-cholesterol; TG = Triglycerides.

### Hardy-Weinberg equilibrium

The genotype distribution was in Hardy-Weinberg equilibrium in both case group and control group (both P > 0.05, data not shown).

### Genotype and allele frequency distributions

As shown in Table 2, for the -75 G > A SNP of apoAI gene, AA genotype frequency (0.501) was significantly higher in patients with osteonecrosis than that in control (0.462) subjects (*P* <0.001), GA genotype frequency (0.170) was significantly lower than that in the control (0.310) group (*P* <0.0001). The genotype and allele frequency distribution in EcoR I, Xba I and 3′-VNTR of apoB gene were also shown in Table 2. For the EcoR I, Xba I and 3′-VNTR of apoB gene, E + E+, X-X- and SS genotypes were more common in both osteonecrosis group and control group. However, for these three loci, the genotype and allele frequencies distribution between the two groups showed no significant difference (all *P* > 0.05).

**Table 2:** **Distributions of ApoAI and ApoB genotypes**

SNPs	Allels (1/2)	Groups	Genotypes (n, %)			***P*** value	Allele (n, %)		OR (95% CI)	***P*** value
			1/1	1/2	2/2		1	2		
-75G > A	G/A	Case	215 (0.501)	73 (0.170)	141 (0.329)	<0.0001	503 (0.586)	355 (0.414)	3.9321 (3.0847 ~ 5.0123)	<0.0001
		Control	170 (0.462)	114 (0.310)	84 (0.228)		624 (0.848)	112 (0.152)		
EcoR I	E+/E-	Case	391 (0.911	38 (0.089)	-	0.151	820 (0.956)	38 (0.044)	0.7288 (0.4667 ~ 1.1381)	0.163
		Control	324 (0.880)	44 (0.120)	-		692 (0.940)	44 (0.060)		
Xba I	X-/X+	Case	394 (0.918)	35 (0.082)	-	0.153	823 (0.959)	35 (0.041)	0.7209 (0.4541 ~ 1.1445)	0.164
		Control	327 (0.889)	41 (0.111)	-		695 (0.944)	41 (0.056)		
3′-VNTR	S/B	Case	336 (0.783)	93 (0.217)	-	0.306	765 (0.892)	93 (0.108)	1.1752 (0.8463 ~ 1.6318)	0.335
		Control	299 (0.813)	69 (0.187)	-		667 (0.933)	69 (0.067)		

1: Represent common allele; 2: Represent rare allele.

In osteonecrosis patients, the OR of A allele was 3.932 (95% CI: 3.0847 ~ 5.0123), which suggested that subjects carrying A allele of promoter region -75 G > A of ApoAI gene had higher susceptibility to osteonecrosis than subjects carrying G allele.

In addition, age and sex adjusted intergenotypic variations in lipid, lipoprotein and apolipoprotein A-I levels with respect to apoAI-75 G/A polymorphism has been summarized in Table 3**.** Rare A allele carriers were associated with lower levels of HDL in both osteonecrosis patients and control subjects (both *P* < 0.0001). Likewise, A allele carriers had lower levels of apoA-I as compared to GG individuals in the osteonecrosis patients group and control group (P < 0.0001).

**Table 3:** **Comparison of lipids levels between each genotype**

Parameters	Groups	GG	GA/AA	P*	P**
TC (mg/dl)	Case	4.9 ± 2.2 (n = 215)	4.7 ± 2.3 (n = 214)	0.358	0.765
	Control	4.7 ± 2.4 (n = 170)	4.6 ± 2.2 (n = 198)	0.677	0.543
LDL-C (mg/dl)	Case	2.8 ± 1.1	2.6 ± 1.3	0.086	0.077
	Control	2.5 ± 1.2	2.3 ± 1.4	0.146	0.714
HDL-C (mg/dl)	Case	1.7 ± 0.9	1.3 ± 0.7	<0.0001	<0.0001
	Control	1.6 ± 1.0	1.4 ± 0.8	<0.0001	<0.0001
TG (mg/dl)	Case	2.2 ± 1.0	2.1 ± 0.8	0.254	0.198
	Control	1.8 ± 0.9	1.7 ± 1.0	0.317	0.177
ApoA-I (g/l)	Case	0.99 ± 0.13	0.86 ± 0.12	<0.0001	<0.0001
	Control	0.94 ± 0.14	0.85 ± 0.11	<0.0001	<0.0001

Note: TC = Total cholesterol; LDL-C = Low-density lipoprotein-cholesterol; LDL-C = High-density lipoprotein-cholesterol; TG = Triglycerides; ApoA-I = Apolipoprotein A-I; *before adjustment; **after adjustment.

## Discussion

In the present study, we found that in patients with osteonecrosis, AA genotype frequency of promoter region -75 G > A in apoAI gene was significantly higher than that in the control group (*P* <0.0001), the GA genotype frequency was significantly lower than that in the control group (*P* <0.0001). However, we did not find significant differences in EcoR I, Xba I and 3′-VNTR region of ApoB gene between patients and control group. Our finding indicated that genetic polymorphism of ApoAI may be associated with risk of osteonecrosis.

At present, it is believed that the abnormal lipid metabolism and intravascular coagulation composed the main pathogenesis of osteonecrosis [3]. Glucocorticoid can cause lipid metabolism abnormality and produce hyperlipidemia via the body lipid mobilization and the blood lipid inhibition in organization of cells [20]. Hyperlipidemia affected the microcirculation of the femoral head to result in femoral necrosis from multiple links, such as affecting blood coagulation solvent systems, influencing bone fat embolism, and affecting the formation of bone micro-thrombosis [21, 22]. Apo AI is the major component of high-density lipoprotein protein which can regulate the high-density lipoprotein metabolism and cholesterol transport in plasma via regulating enzyme activity, receptor activity and diacylglycerol/protein kinase C (DG / PKC) pathway. Apo AI gene is located on the long arm of chromosome 11, and has multiple restriction enzyme sites. Previous studies found that at the start site of human Apo AI gene transcription, G/A polymorphism existed at upstream -75 bp regions, this polymorphism was located at GC-rich region in ApoAI gene promoter, and the GC-rich region was the regulatory elements of Apo AI gene transcription. When the sequence changed, the gene transcription and expression will be affected, and Apo AI synthesis would be affected. ApoAI gene structure changed and affected the molecular structure of Apo AI and the generation and the release rate into the blood. Thereby it will affect the anti-atherosclerotic function of the blood ApoAI and high-density lipoprotein cholesterol levels, the lipid metabolism disorders would easily occur. Al-Bustan et al. reported a significant association of the APOAI -75 G > A polymorphism with increased serum LDL-C. Multivariate analysis showed that APOAI -75 G > A was an independent predictive factor when controlling for age, sex and BMI for both LDL-C and TC levels. In the present study, our data revealed that ‘A’ allele carriers were associated with lower HDL levels as compared to GG homozygous in patients (18.8% lower, *P* < 0.0001) and control subjects (16.6% lower, *P* < 0.0001). Also, apoA-I levels were found to be lower in GA and AA individuals as compared to GG in the patients (13.1% lower, *P* < 0.0001) and control subjects (9.6% lower, *P* < 0.0001).

ApoB was the component of lipoprotein (a), chylomicrons, very low density lipoproteins and intermediate density lipoprotein, it was the only structural protein of LDL. It mainly combined with cholesterol to form the low-density lipoprotein and recognized the low-density lipoprotein receptor, which played an important role in the process of transportation and metabolism of lipids. The apoB gene polymorphism can affect the plasma TC, LDL and ApoB levels [23]. Previous studies indicated that Xba I, EcoR I and 3′-VNTR in ApoB gene were associated with lipid metabolism and related diseases [24, 25]. However, in the present study, we did not found association of these SNPs in ApoB gene and osteonecrosis.

Although we found a positive association between -75 G > A polymorphism and osteonecrosis, the present study was limited by the relatively small sample size. This may have led to weak statistical significance and wide CIs when estimating odds ratios. In addition, we did not perform functional study of these SNPs, which may be another limitation of our study.

## Conclusion

In conclusion, this study showed that -75 G > A polymorphism in ApoAI gene may be associated with osteonecrosis in Han Chinese population.
